# Draft Genome Sequences of Multidrug-Resistant Campylobacter jejuni Strains Isolated from Chickens in Central China

**DOI:** 10.1128/MRA.01303-19

**Published:** 2020-01-02

**Authors:** Yiluo Cheng, Wenting Zhang, Qin Lu, Guoyuan Wen, Qingping Luo, Huabin Shao, Tengfei Zhang

**Affiliations:** a Key Laboratory of Prevention and Control Agents for Animal Bacteriosis, Institute of Animal Husbandry and Veterinary, Hubei Academy of Agricultural Sciences, Wuhan, China; University of Maryland School of Medicine

## Abstract

Campylobacter jejuni is a major foodborne pathogen that plays an important role in spreading drug resistance. We report the draft genome sequences of two multidrug-resistant C. jejuni isolates which contained similar mutations in the CmeR box. This will improve the understanding of C. jejuni antimicrobial resistance and genetic characteristics.

## ANNOUNCEMENT

Campylobacter jejuni is a major foodborne pathogen that causes bacterial gastroenteritis in humans (1–3). Poultry has been recognized as the most important reservoir of this pathogen. Recently, the antibiotic resistance of C. jejuni has been increasing due to the prophylactic or therapeutic application of antimicrobials in animal husbandry (4). The availability of genomic sequences would provide valuable knowledge for understanding the antimicrobial resistance and genetic characteristics of C. jejuni.

In 2014, two C. jejuni strains, XZJ48 and XZJ52, were isolated from anal swabs from chickens in wet markets in Hubei Province, China. The C. jejuni strains were cultured at 42°C in Bolton broth containing *Campylobacter* growth supplements and *Campylobacter* selective supplements (Oxoid, England), under microaerobic conditions. Antimicrobial susceptibility was analyzed according to the Clinical and Laboratory Standards Institute guidelines (5), and the results showed that XZJ48 and XZJ52 were resistant to at least six antimicrobial agents, including cefoperazone (64 mg/liter for XZJ48 and 128 mg/liter for XZJ52), tetracycline (64 mg/liter for XZJ48 and XZJ52), sulfamethoxazole (8 mg/liter for XZJ48 and XZJ52), ciprofloxacin (128 mg/liter for XZJ48 and XZJ52), erythromycin (16 mg/liter for XZJ48 and 512 mg/liter for XZJ52), and clindamycin (8 mg/liter for XZJ48 and XZJ52).

To isolate the genome DNA, the strains were cultured for 24 h at 42°C in Bolton broth containing *Campylobacter* growth supplements, under microaerobic conditions. Genomic DNA was extracted using the MiniBEST universal genomic DNA extraction kit (TaKaRa, Dalian, China), according to the manufacturer’s instructions. The sequencing library was generated using the TruSeq DNA sample preparation kit (Illumina, San Diego, CA). Sequencing of C. jejuni XZJ48 and XZJ52 was performed using the TruSeq SBS kit (Illumina), on an Illumina MiSeq PE150 platform (run configuration, 2 × 150 bp). The paired-end reads were adapter trimmed and assembled with SOAPdenovo v2.04 (6) and GapCloser v1.12 (7). Default parameters were used for all software unless otherwise specified. All relevant sequencing and assembly statistics are summarized in Table 1.

**TABLE 1:** Sequencing and assembly statistics for C. jejuni strain genomes

Strain	No. of reads in raw data	No. of total bases in raw data	No. of reads in clean data	No. of total bases in clean data	Avg coverage (fold)	No. of contigs	No. of bases in all contigs	GC content (%)	*N*_50_ (bp)	*N*_90_ (bp)
XZJ48	15,205,214	2,280,782,100	14,350,745	2,116,736,070	1,156	90	1,831,780	30.76	246,974	92,068
XZJ52	25,601,202	3,840,180,300	24,417,852	3,607,441,610	1,910	110	1,889,087	31.28	188,109	92,149

Multilocus sequence typing was carried out based on the sequences of seven housekeeping genes and showed that the sequence types (STs) of XZJ48 and XZJ52 were ST-7510 and ST-7512, respectively (8). ST-7510 and ST-7512 belong to the ST-353 clonal complex, which is the most common clonal complex worldwide (9). Resistance genes in strains XZJ48 and XZJ52 were screened using ARG-ANNOT (10), which showed that both of them contained the tetracycline resistance gene *tetO* and the fluoroquinolone-resistance-related Thr-86-Ile substitution in GyrA (11) but not other known resistance genes or mutations (12, 13). The CmeABC efflux pump has been characterized as contributing to antimicrobial resistance in C. jejuni; it is negatively regulated by CmeR binding to the CmeR box (TGTAATATTTATTACA [inverted repeats are underlined]) in the promoter of the *cmeABC* operon (14, 15). Both XZJ48 and XZJ52 were found to contain an adenine point deletion between two repeats of the CmeR box (TGTAAT–TTTATTACA), and this point deletion was confirmed by PCR and sequencing, as reported previously (16, 17). In addition, this point deletion was further identified, by PCR and sequencing, in 11 multidrug-resistant isolates of 143 *Campylobacter* isolates in our laboratory. As reported previously, a mutation in the CmeR box could cause derepression and overexpression of *cmeABC* and then accelerate the excretion of antimicrobial agents in C. jejuni (17). We think that the adenine deletion in the CmeR box is responsible for multidrug resistance in these two C. jejuni isolates, and we will further investigate the role of this point deletion in multidrug resistance.

### Data availability.

The whole-genome shotgun sequencing projects were deposited in GenBank under accession numbers SZOP00000000 (XZJ48) and SZWG00000000 (XZJ52). The raw sequence data were deposited under SRA accession numbers SRR10297828 (XZJ48) and SRR10297827 (XZJ52).
